# Supplementary material for: Dynamic transcriptomic profiles of zebrafish gills in response to zinc supplementation
Source: BMC Genomics. 2010 Oct 11;11:553. doi: 10.1186/1471-2164-11-553 (PMC3091702; doi:10.1186/1471-2164-11-553)
Supplement: Additional file 2 — Interactive Direct Interaction Network representing the molecular interactions between zinc, copper, iron, calcium and proteins encoded by transcripts changed by zinc supplementation. Mini web-site containing index.html and hyperlinked pages in subdirectory describing a Direct Interaction Network automatically generated based on curated interactions contained within the proprietary PathwayArchitect database. Ovals represent proteins and the circles symbolize metal ions. Objects are coloured by their abundance in zebrafish at the time-point they were significantly different from the control is a scale from -4 fold (dark green) to +4 fold (dark red). Where significant differences were found at more than one time-point, the colour overlay shows expression at the first instance. Dark blue squares denote 'binding', and light blue squares 'expression'; green squares stand for 'regulation', green diamonds for 'metabolism', and green circles for 'promoter binding'. Arrow heads indicate directionality of the interaction where annotated. All nodes and edges can be further interrogated by selecting the relative area of the image. [file 1471-2164-11-553-S2.zip › PathwayArchitect Zn xs DIN/754140.html]

# REGULATION:

|  |  |
| --- | --- |
| Type | REGULATION |
| Effect | Negative |


---

|  |  |
| --- | --- |
| Score | 0 |


---

|  |  |
| --- | --- |
| Reference Count | 5 |


---

|  |  |
| --- | --- |
| Mechanism | Unknown |


---

|  |  |
| --- | --- |
| Reference:0 || Sentence | "Of 7 iron chelators, only deferriprone (L1) markedly decreased Tf uptake." |
| PMID | 10865941 |
| Year | 2000 |
| Species | Mouse |
| Journal | Endothelium |
| RefScore | 0 |
| Source | PArchNLP |
  |
|  |  |


---

|  |  |
| --- | --- |
 Reference:1 || Sentence | "Plasma Tf concentrations were lower in +/hpx mice, plasma Tf nearly doubled and liver Tf was only 50% of normal in response to iron deficiency." |
| PMID | 9850571 |
| Year | 1998 |
| Species | Mouse |
| Journal | Biometals |
| RefScore | 1 |
| Source | PArchNLP |
  ||  |  |


---

|  |  |
| --- | --- |
 Reference:2 || Sentence | "Iron administration to transgenic mice resulted in a significant decrease of transferrin-directed CAT enzyme activity and CAT protein in liver, but no significant decrease in human transferrin-CAT mRNA levels." |
| PMID | 8490018 |
| Year | 1993 |
| Species | Human |
|  | Mouse |
| Journal | Biochemistry |
| RefScore | 1 |
| Source | PArchNLP |
  ||  |  |


---

|  |  |
| --- | --- |
 Reference:3 || Sentence | "The present findings demonstrate that iron distribution in the brain is changed by transferrin deficiency and that iron abnormally accumulates in the brain of HP mice." |
| PMID | 11532431 |
| Year | 2001 |
| Species | Mouse |
| Journal | Brain Res |
| RefScore | 2 |
| Source | PArchNLP |
  ||  |  |


---

|  |  |
| --- | --- |
 Reference:4 || Sentence | Low-dose continuous iron therapy leads to a positive iron balance and decreased serum transferrin levels in chronic haemodialysis patients. |
| Year | 2004 |
| PMID | 15004263 |
| Species | Human |
| Journal | Nephrol Dial Transplant |
| RefScore | 2 |
| Source | PArchNLP |
  |


---

|  |  |
| --- | --- |
